# Hemangiosarcoma and its cancer stem cell sub-population are effectively killed by a toxin targeted through epidermal growth factor and urokinase receptors

**DOI:** 10.1186/1753-6561-7-S2-P34

**Published:** 2013-04-04

**Authors:** Jill T Schappa, Aric M Frantz, Brandi H Gorden, Erin B Dickerson, Daniel A Vallera, Jaime F Modiano

**Affiliations:** 1 Veterinary Clinical Sciences, University of Minnesota, Minneapolis, MN 55455, USA; 2 Masonic Cancer Center, University of Minnesota, Minneapolis, MN 55455, USA; 3 Therapeutic Radiology, University of Minnesota, Minneapolis, MN 55455, USA

## Background

Targeted toxins have the potential to overcome intrinsic or acquired resistance of cancer cells to conventional cytotoxic agents. We hypothesized that EGFuPA-toxin, a bispecific ligand-targeted toxin consisting of a deimmunized *Pseudomonas* exotoxin conjugated to epidermal growth factor (EGF) and urokinase (uPA), would efficiently target and kill cells derived from canine hemangiosarcoma (HSA), a highly chemotherapy resistant tumor, as well as cultured hemangiospheres, used as a surrogate for cancer stem cells (CSC).

## Materials and methods

We evaluated EGFuPA-toxin activity in four HSA cell lines (Emma, Frog, DD-1, and SB), using a feline mammary carcinoma cell line (K12) and a human T-cell leukemia line (Jurkat) as controls. Hemangiospheres were grown under serum-free low adherence conditions to enrich cancer stem cells. Cytotoxicity was determined using the CellTiter96 AQueous viability assay. Specificity for cells expressing cognate receptors was confirmed using neutralizing antibodies and competitive binding assays. Relative receptor expression in target cell lines was verified using flow cytometry.

## Results

EGFuPA-toxin showed cytotoxicity in each of the HSA cell lines tested at concentrations ≤100 nM that was dependent on specific ligand-receptor interactions. Monospecific targeted toxins also killed HSA cells; in this case, a “threshold” level of EGFR expression appeared to be required to make cells sensitive to monospecific EGF-toxin, but not to monospecific uPA-toxin. The IC50 of CSCs was higher by approximately two orders of magnitude compared to non-CSCs, but these cells were still sensitive to EGFuPA-toxin at nanomolar (*i.e.*, pharmacologically relevant) concentrations.

## Conclusions

Our results support the use of these toxins to treat chemoresistant tumors such as sarcomas, including those that conform to the cancer stem cell model. Our results also support the use of companion animals with cancer for further translational development of these cytotoxic molecules.

## Financial support

Supported by NIH grants R01 CA036725 (DAV) and P30 CA077598 (Masonic Cancer Center, U of M), AKC CHF 1131, NCCF DM06CO-002, and MAF D10CA-501 (JFM). DI is recipient of MAF First Award D12CA-302. JTS was supported through an individual HHMI/BWF medical research fellowship.

